# Artificial Plantation Responses to Periodic Submergence in Massive Dam and Reservoir Riparian Zones: Changes in Soil Properties and Bacterial Community Characteristics

**DOI:** 10.3390/biology10080819

**Published:** 2021-08-23

**Authors:** Jiajia Li, Lijuan Li, Muhammad Arif, Dongdong Ding, Xin Hu, Jie Zheng, Zhongxun Yuan, Changxiao Li

**Affiliations:** Key Laboratory of Eco-Environments in the Three Gorges Reservoir Region (Ministry of Education), Chongqing Key Laboratory of Plant Resource Conservation and Germplasm Innovation, College of Life Sciences, Southwest University, Chongqing 400715, China; ljj133888@email.swu.edu.cn (J.L.); zxc124487991@email.swu.edu.cn (L.L.); muhammadarif@swu.edu.cn (M.A.); dingdongdong@email.swu.edu.cn (D.D.); sthuxin@email.swu.edu.cn (X.H.); jiezheng@email.swu.edu.cn (J.Z.); yuanzhongxun@email.swu.edu.cn (Z.Y.)

**Keywords:** Three Gorges Dam Reservoir, woody and herbaceous plants, soil nutrients and enzyme, soil microorganism, different growth period

## Abstract

**Simple Summary:**

This study focuses on plants in riparian zones that are very vulnerable due to water stress and anthropogenic disturbances, which are particularly important regarding their ecological and environmental role. Although plants and microbiome interactions are necessary for plant nutrient acquisition, relatively little is known about the responses of roots, bulk, and rhizosphere soil microbial communities of different artificial vegetation types in riparian areas of massive dams and reservoirs. Therefore, this study aims to assess the responses of woody and herbaceous plants in the riparian zones of the Three Gorges Dam Reservoir, China. Results revealed that the weight of dominant soil bacteria in different periods, including *Proteobacteria*, *Acidobacteria*, *Actinobacteria*, *Chloroflexi*, and *Cyanobacteria*, was higher, and their composition was different in the rhizosphere, bulk soil, and endophyte. In the soil co-occurrence networks, the weight of soil physical properties was higher than chemical properties in the early emergence stage. The current study provides knowledge about bacteria in bulk, rhizosphere soils, and within roots in different emergence phases. Additionally, these results provide valuable information to inoculate the soil with key microbiota members by applying fertilizers, potentially improving plant and soil production and health.

**Abstract:**

Plant and microbiome interactions are necessary for plant nutrient acquisition. However, relatively little is known about the responses of roots, bulk, and rhizosphere soil microbial communities in different artificial vegetation types (woody and herbaceous) in riparian areas of massive dams and reservoirs. Therefore, this study aims to assess such responses at elevations of 165–170 m a.s.l. in the riparian zones of the Three Gorges Dam Reservoir, China. The samples were collected containing the rhizosphere soil, bulk soil, and roots of herbaceous and woody vegetation at different emergence stages in 2018. Then, all the samples were analyzed to quantify the soil properties, bacterial community characteristics, and their interaction in the early and late emergence phases. In different periods, the weight of dominant soil bacteria, including *Proteobacteria*, *Acidobacteria*, *Actinobacteria*, *Chloroflexi*, and *Cyanobacteria*, was higher, and their composition was different in the rhizosphere, bulk soil, and endophytes. Moreover, the soil co-occurrence networks indicated that the weight of soil physical properties was higher than chemical properties in the early emergence stage. In contrast, the weight of chemical properties was relatively higher in the late emergence stage. Furthermore, the richness and diversity of the bacterial community were mainly affected by soil organic matter. This study suggests that these herbaceous and woody vegetation are suitable for planting in reservoir areas affected by hydrology and human disturbance in light of soil nutrients and soil microbial communities, respectively. Additionally, these results provide valuable information to inoculate the soil with key microbiota members by applying fertilizers, potentially improving plant health and soil production.

## 1. Introduction

The soil has substantial heterogeneity in the riparian zones of dams [1], and its nutrient levels depend on many factors. The riparian soil is affected by such key factors as water stress, anthropogenic disturbance, and vegetation cover [2,3]. The flora of the reservoir is usually affected by the seasonal water level regulations [4]. As a result, soil types in the riparian zone present seasonal changes and decrease natural vegetation diversity and biomass [5]. For example, water stress reduced the riparian vegetation’s total biomass accumulation, nitrogen, and phosphorus uptake, seriously affecting plant growth and development [6]. Water content changes in the riparian soils could potentially affect plant-microbial interactions already in existence under different water conditions [7]. Similarly, anthropogenic disturbances deteriorate the riparian zones [8], often through agricultural non-point source pollution and waste discharge, leading to abnormal changes in soil pH, nitrogen, organic matter, and iron content, and even other soil properties [9]. Additionally, agricultural systems, land use, and other factors also significantly impact riparian health [8]. Former studies have found that human activities also affect aboveground biomass, dead matter, underground biomass, carbon storage, and other indicators [10]. Therefore, the soil in the riparian zone is affected by these hydraulic gradients and anthropogenic disturbance, which would change the balance of vegetation-soil-microorganisms.

Soil microorganisms are sensitive indicators of microenvironmental changes in soil [11]. In the vegetation-soil-microbial cycle system, soil microorganisms participate in the soil nutrient cycle and energy transfer. They also indirectly affect the growth of aboveground vegetation by improving soil aeration, decomposition, and increasing organic matter content, thus directly impacting the function of the soil ecosystem [12,13]. Studies have reported that microorganisms have various properties, directly or indirectly increasing the soil content of nitrogen, phosphorus, potassium, iron, and other minerals [9,10]. It can also promote mineral utilization for plants to grow, develop, and increase crop yield [14]. Furthermore, microorganisms in the rhizosphere and the root plane contribute to plant acquisition of minerals, organic matter, and other small-molecule metabolites. The interaction between microorganisms and plants can directly or indirectly promote plant growth, provide biological control over biotic and abiotic stresses, and improve biogeochemical cycles in natural ecosystems [15,16]. Moreover, substances such as organic matter, auxin, etc., when transported from plants to the soil, can also affect soil microorganism activity [17]. When soil organic matter is mineralized, it can provide a food source for participating in microbial nutrient cycling [18,19]. In addition, microbial activity is also related to other soil properties, such as soil pH, nitrogen, and phosphorus [20].

In the riparian zones, plant roots also play a crucial role in stabilizing riparian soil ecosystems. Plant roots can form a network during plant growth and development to improve soil retention capacity through winding and reinforcement [21]; on the other hand, plant roots can secrete and form many cementing substances, using chemical action to bond soil particles and affect soil properties and microflora [22]. Literature has proven that riparian vegetation can also regulate soil nutrients through absorption, decomposition, and deposition of alluvial materials [23]. It can indirectly control the rhizosphere microbe through the rhizosphere secretions of secondary plant metabolites [24]. Additionally, such compounds as nematocides and flavonoids, which are conducive to microorganisms establishing a symbiotic relationship or resistance to pests and diseases, release sediments, including low-molecular compounds (sugars, amino acids, organic acids, etc.), polymeric sugars (mucus, etc.), and root margin cells [25,26]. Dead root cap cells are used as carbon sources by soil microorganisms. While serving as carbon sources and nutrients for microbial growth, root exudates and rhizosphere sediments will also affect soil properties. Therefore, the vegetation-soil-microbe balance is essential in the riparian zones under anthropogenic disturbance and flooding stress [27].

With the implementation of the Three Gorges Dam (TGD) Project in 2008, the water level of the reservoir fluctuated from 145 m a.s.l. in summer (May to September) to 175 m a.s.l. in winter (October to April) [28], resulting in the formation of the riparian zones with a total area of 350 km^2^ in the reservoir [29]. This unique hydrological rhythm makes plants and soil suffer from long-term water flooding stress [30]. However, riparian zones above 167.5 m a.s.l. are mainly affected by human disturbance [31], while below 167.5 m a.s.l. are mainly affected by hydrological disturbance. Thus, the changes in soil properties along an elevation gradient from 160 m to 170 m a.s.l. are caused by both water flooding stress and human disturbance [1,29]. Yet, flooding stress and human disturbance have significantly degraded native plants’ habitats, reduced riparian biodiversity and ecological function, and brought other environmental problems [1,8]. Still, many studies have shown that *Cynodon dactylon*, *Hemarthria altissima*, *Taxodium distichum*, and *Salix matsudana* can adapt to the habitat of this area to improve soil quality [32] and are now widely planted in this area. In the riparian zones of the TGD region, many studies have focused on the changes in soil properties and plant community composition under the altitudinal distribution pattern [33]. However, soil microhabitat changes caused by plant root activity are still poorly understood in the riparian zones affected by both human disturbance and flooding stress. 

Therefore, the responses of microbial communities in roots, bulk, and rhizosphere soil under different artificial vegetation plantations in the riparian zones of the TGD region in different periods were investigated in this research. Such studies are critical for managers to understand those biotic and abiotic interactions in order to maintain environmental stability and provide valuable information for inoculating the soil with important microbiota members. Moreover, applying fertilizers can be handy for further vegetation restoration in the affected riparian zones from anthropogenic disturbance and flooding stress. The following questions are thus hypothesized in our research: in different emergence periods, (1) how do the roots, bulk, and rhizosphere soil microbial community characteristics respond to artificial vegetation plantations; (2) which soil properties have a more significant influence on the bacterial microbial community; and (3) what is the complexity of the co-occurrence networks between soil properties and the soil bacterial community?

## 2. Materials and Methods

### 2.1. Site Description

**Figure 1:**
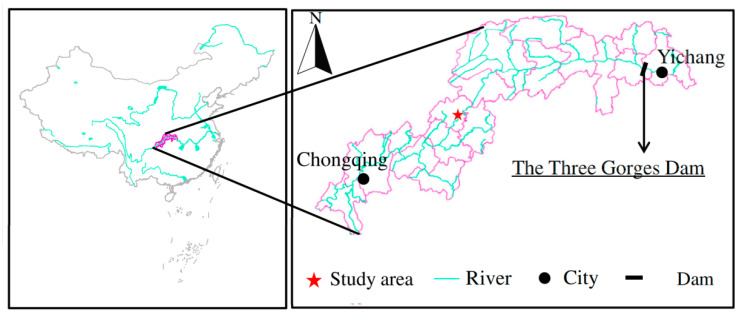
Location of sample site in the riparian zone of the Three Gorges Dam, China.

Our study was conducted in the riparian zone of the Ruxi River in Zhong County (107°32′~108°14′ E, 30°03′~30°35′ N), Chongqing, China (Figure 1). This region is part of the subtropical southeast monsoon zone, and the annual mean temperature is 18.2 °C. The frost-free period is 341 d. The sunshine duration remains at 1327.5 h with a daily illumination rate of 29%. The total solar radiant energy is 3.5 × 10^5^ J·cm^−2^ in the area. The annual precipitation is 1200 mm, and there is a relative humidity of 80%. This area is a typical hilly landform with an average slope of 20~25°. The primary soil type is calcareous purple soil (Regosols in FAO Taxonomy). Due to the unique hydrological changes in the reservoir area, water loss and soil erosion in the hydro-fluctuation zone are more serious [34]. In this study, perennial woody plants (*S*. *matsudana* and *T*. *distichum*) and perennial herbs (*C*. *dactylon* and *H*. *altissima*) were selected as experimental materials. The woody and herbaceous species selected in this experiment were planted in the vegetation restoration demonstration area in the mid-to-high-altitude area. Field survey results showed that after repeated flooding for six years, these four leading plant species still displayed rapid growth and development, recovered rapidly from the outcrop stage of the fluctuation zone, and quickly occupied a particular spatial ecological niche [35].

### 2.2. Soil Sampling

Soil samples were collected in July (early emergence phase-T1) and September (late emergence phase-T2) of 2018, when the plants in the riparian zone (165–170 m a.s.l.) were growing well. According to the growth and distribution of plants, the S-shaped sampling zone was delineated in the riparian zone. Three 1 m × 1 m quadrats were randomly set on the S-shaped transect in the herbaceous vegetation. Then, in each quadrat, five soil blocks (15 cm × 15 cm × 20 cm) were placed in the shape of a quincunx. Herbaceous rhizosphere soil was collected by referring to the shaking method of Riley [36], while bulk soil samples were collected by the quartering method. Under woody vegetation, three plants with similar growth were randomly selected for destructive sampling. Woody rhizosphere soil samples were collected using the clod method [37,38]. After removing visible stone and plant residues from the soil surface, four sections (20 cm long, 20 cm wide, and 20 cm high) of the trunk were excavated with a shovel along the east, south, west, and north directions of the trunk, and soil samples were collected. The soil within 0~4 mm of the root system is regarded as the rhizosphere soil [39]. The roots mixed in the soil were picked out. The soil samples from four directions were mixed into a frozen foam box and immediately transported back to the laboratory for subsequent analysis and determination of experimental indicators.

### 2.3. Determination Method

The rhizosphere and bulk soil samples were divided into three parts. One part of the soil sample was naturally air-dried. After grinding, the fine roots were separated, and then the soil was used to determine soil physical and chemical properties. Some soil samples were stored at 4 °C for the determination of soil enzyme activity. The other parts were stored at −80 °C for subsequent extraction of soil bacterial DNA.

#### 2.3.1. Determination of Soil Nutrients

The electrode potential method was used to measure the soil pH value (1:2.5 ratio of soil to water). The external heating method with potassium dichromate determined the soil organic matter (SOC). An elemental (Germany) analyzer computed total soil nitrogen (TN) (Elementar, Hanau, Germany). The alkaline hydrolyzed nitrogen diffusion method quantified the soil alkali hydrolyzed nitrogen (AN). The Mo-Sb colorimetric method calculated total phosphorus (TP) and available phosphorus (AP). The inductively coupled plasma emission spectrophotometry (ICP-OES) determined the total potassium (TK) and available potassium (AK) [40].

The drying method measured soil water content (SWC), and the ring knife method measured bulk density (BD). Soil porosity (SP) was calculated from soil density and measured bulk density [32]. Using a REDOX potentiometer, we measured soil temperature (ST) and oxidation-reduction potential (ORP).

The 3,5-Dinitrosalicylic acid colorimetry determined sucrose (INV) activity, expressed as 1 g glucose mass (mg) generated by 1 g soil cultured at 37 °C for 24 h. The urease (URE) activity was determined using sodium phenol colorimetry, expressed as the mass (mg) of NH_3_-N generated by 1 g soil cultured at 37 °C for 24 h. The acid phosphatase (ACP) activity was determined using the colorimetric method of phenyl disodium phosphate, which was expressed as the mass of phenol released in 1 g soil after 24 h (mg). Each sample had three replicates and was set up with no matrix control and soilless control. 

#### 2.3.2. Soil Microbial Community Determination

Sample DNA extraction was conducted using the Mobo Power Soil Isolation Kit according to the instructions. The concentration of the extract was determined by 1.2% agarose gel electrophoresis, and the extracted DNA was cryopreserved at −20 °C. The bacterial 16S V4–V5 region universal primer was used to amplify each sample three times and mix the same sample amplification products. The primers used were 515F (5′-GTGCCAGCMGCCGCGG-3′) and 907R (5′-CCGTCAATTCMTTTRAGTTT-3′). PCR products were purified by gel-cutting, qubit quantification, and equal-molar mixing to establish the sequencing library. The soil bacterial community diversity was analyzed by IlluminaHiSeq sequencing. The coarse quality sequences of the sequencing machine were split according to BarcodeMisMatch = 0. The double-ended sequences were split by Flash software, and the front-end primers were removed by Cutadapt software. The quality control of the sequences was carried out according to the quality score Q20 to obtain high-quality sequences [41]. Then the UPARSE algorithm was used to cluster OTUs of high-quality sequences and obtain representative sequences. In the clustering process, RDP gold. fa was used as a template to remove chimeric sequences, and a RDP classifier was used to annotate the OTU representative sequences [42]. According to the number of sequences contained in each sample, the OTU table was randomly sampled, and the sampling results were used for downstream analysis.

### 2.4. Statistics Analysis

A one-way analysis of variance (ANOVA) was performed for multiple comparisons to determine the significant differences between soil nutrient properties and between soil enzyme activities. Then, a two-tailed unpaired test was performed. All statistical tests performed using SPSS 23.0 software (SPSS Inc., Chicago, IL, USA) concluded their significance at *p* < 0.05. Gephi software was used to establish the co-occurrence networks of soil nutrients and microorganisms. The R platform (version 4.0.3) ran the following analyses and generated plots using the “ggplot2” package. To compare the difference in bacterial community structure with the overall difference in plant-soil nutrient content, principal coordinate analysis (PCOA, based on the Bray-Curtis distance measure) and principal component analysis (PCA, based on the Euclidean distance measure) were performed. Additionally, Pearson correlation analysis was calculated in the “Corrplot” package to reveal the correlation between the selected soil bacterial community abundance and soil nutrients. The “RandomForest” package was used to evaluate the weight of soil properties on microbial community diversity and richness. 

## 3. Results

### 3.1. The Soil Physical and Chemical Properties in Different Periods

When compared between different emergence periods (Table 1), all the soil physical properties in *C*. *dactylon* were significantly different (* *p* < 0.05, ** *p* < 0.01), except SWC. Conversely, all the soil physical properties in *S*. *matsudana* showed no significant difference, except for ST (** *p* < 0.01). There were significant differences in the ST and SWC in *H*. *altissima* and *T*. *distichum* (* *p* < 0.05, ** *p* < 0.01, respectively). The variation trend in different periods of soil physical properties in *C*. *dactylon* and *H*. *altissima* was consistent; likewise, *T*. *distichum* and *S*. *matsudana* were consistent.

**Figure 2:**
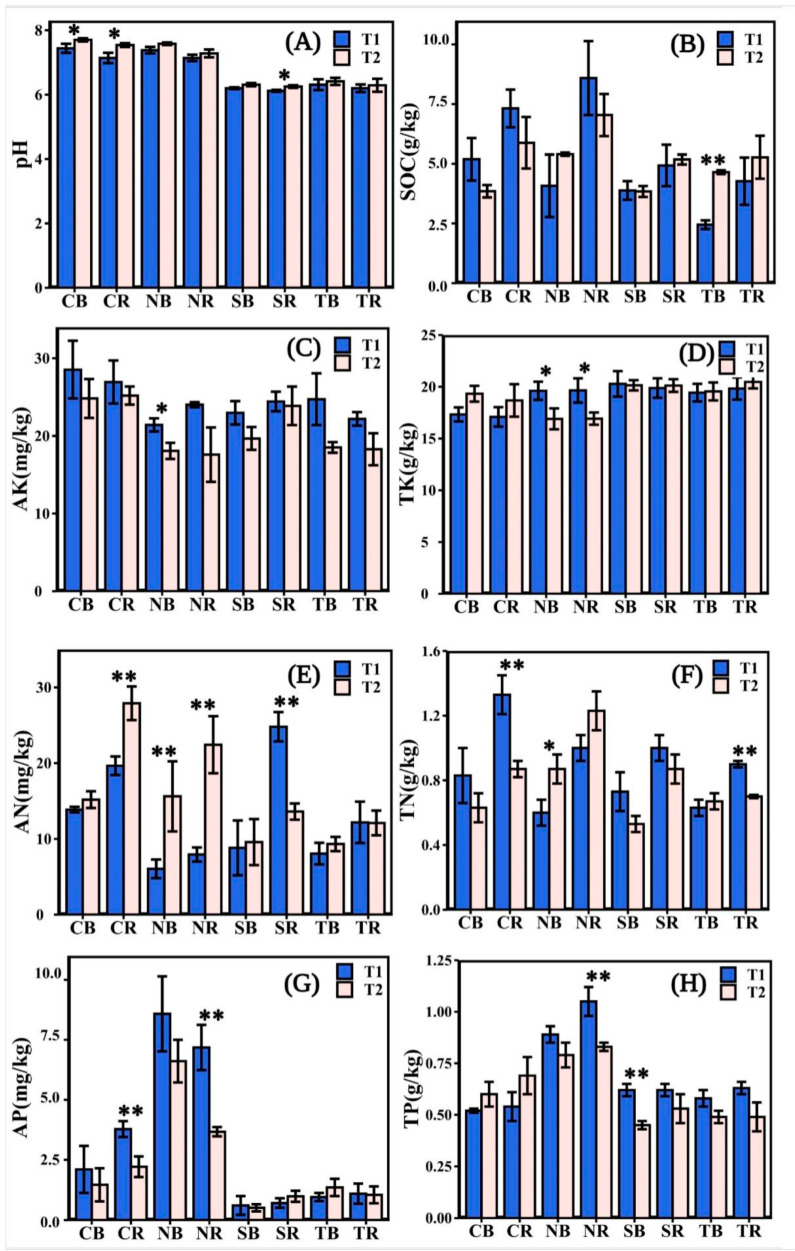
The soil chemical properties measured in different emergence phases (T1 for early phase and T2 for late phase) within the Three Gorges Dam Reservoir, China. The values of pH, soil organic contents (SOC), available potassium (AK), total potassium (TK), alkali hydrolyzed nitrogen (AN), total nitrogen (TN), available phosphorus (AP), total phosphorus (TP) available in the rhizosphere and bulk soil measured for selected plants in (**A**–**H**). Note: bars indicate significant differences at *p* < 0.01 (**) or *p* < 0.05 (*) according to the paired *t*-tests. Abbreviations in the figure define as bulk soil (B), rhizosphere soil (R), *Cynodon dactylon* (C), *Hemarthria altissima* (N), *Salix matsudana* (S), *Taxodium distichum* (T).

The soil pH and TK in the four-plant species did not change significantly, but the other indicators changed to various degrees (Figure 2). The soil pH was lower in T1 than in T2 (Figure 2A). The SOC of herbaceous plants was higher in T1 than in T2, except for NB, while the SOC of woody plants was contradictory (Figure 2B), and the SOC (8.59 g kg^−1^) of NR was relatively higher. Overall, AK in the rhizosphere and bulk soil of all plants was lower in T2 than in T1 (Figure 2C). The AK (28.53 mg kg^−1^) of CB was higher in T1. The TK of *C*. *dactylon* was lower in T1 than in T2 (Figure 2D); TK of *H*. *altissima* was significantly higher in T1 than in T2 (* *p* < 0.05). The TK of woody plants fluctuated slightly at different stages (Figure 2D). In T2, the AN (27.91 mg kg^−1^) of CR was higher than in other plants. The AN of herbaceous plants was significantly higher at T2 than at T1 (Figure 2E) (** *p* < 0.01). The TN (1.33 g kg^−1^) of CR in T1 was higher than that of others (Figure 2F). The content of AP in the rhizosphere soil of herbaceous plants was significantly higher at T1 than at T2 (Figure 2G) (** *p* < 0.01), and the AP (8.58 mg kg^−1^) of NB in T1 was higher than that of others. TP was lower in T2 than in T1 (Figure 2H), except for *C*. *dactylon*. The TP (1.05 g kg^−1^) of NR in T1 was higher than that of others. In general, the soil nutrient contents of *C*. *dactylon* and *H*. *altissima* were higher among the four plants.

**Figure 3:**
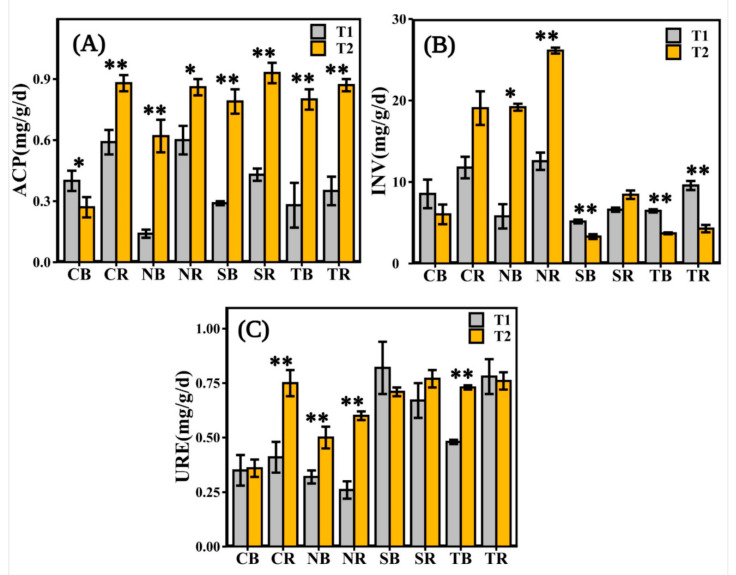
The soil enzyme activities measured in different emergence phases (T1 for early phase and T2 for late phase) within the riparian zones of the Three Gorges Dam Reservoir, China. The values of acid phosphatase (ACP), invertase (INV), and urease (URE) were available from the rhizosphere and bulk soil of selected plants in (**A**–**C**). Note: bars indicate significant differences at *p* < 0.01 (**) or *p* < 0.05 (*) according to the paired *t*-tests. Abbreviations in the figure define as bulk soil (B), rhizosphere soil (R), *Cynodon dactylon* (C), *Hemarthria altissima* (N), *Salix matsudana* (S), *Taxodium distichum* (T).

The ACP activity was significantly different in all the measured soils in different periods. Except for CB (Figure 3A), ACP enzyme activity was significantly higher in the late emergence phase than in the early emergence phase. The soil INV enzyme activity was substantially different in herbaceous plants and woody plants. The INV enzyme activity of herbaceous plants in the late emergence phase was significantly higher than in the early emergence phase, except for CB and CR (Figure 3B). There was no significant difference in URE activities between the rhizosphere and bulk soils for woody plants in different periods, except for TB (Figure 3C). The URE enzyme activity of herbaceous plants in the late emergence phase was significantly higher than in the early emergence phase, except for CB (Figure 3C). 

**Figure 4:**
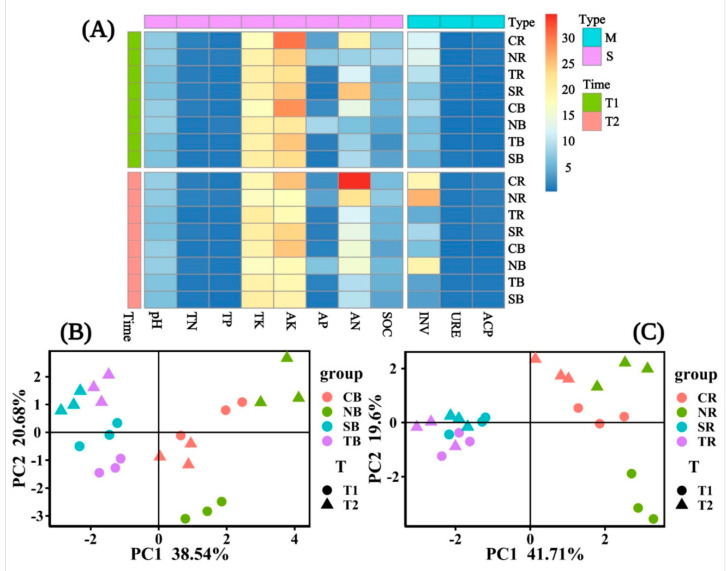
The heat map showed the combinations of soil enzyme (M) and soil properties (S) under different emergence phases (T1 for early phase and T2 for late phase) within the riparian zones of the Three Gorges Dam Reservoir, China (**A**). Principal component analysis (PCA) showed the difference in soil properties in T1 and T2. The factor loading of PCA axes 1 and 2 explained 59.22% and 61.31% of the total variation for the bulk soil and rhizosphere soil in (**B**,**C**), respectively. Note: Abbreviations in the figure define as pH value (pH), available potassium (AK), total potassium (TK), alkali hydrolyzed nitrogen (AN), available phosphorus (AP), soil organic contents (SOC), total nitrogen (TN), total phosphorus (TP), acid phosphatase (ACP), invertase (INV), and urease (URE), bulk soil (B), rhizosphere soil (R), *Cynodon dactylon* (C), *Hemarthria altissima* (N), *Salix matsudana* (S), *Taxodium distichum* (T).

Among the soil properties of the four plants, we observed the general variation of the bulk and rhizosphere soils (Figure 4A). We discovered that the bulk and rhizosphere soil properties of *C*. *dactylon* and *H*. *altissima* differed significantly over time, but *H*. *altissima*’s soil properties varied greatly over time compared to those of other plants (Figure 4B,C). In the bulk soil, the soil properties of woody plants were significantly different in different periods (Figure 4B), but there was no significant difference in rhizosphere soil (Figure 4C). Overall, the differences in the bulk soil properties of selected herbaceous and woody plants were significant. Only herbaceous plants showed significant differences between time periods in the rhizosphere. In different periods, the variations in the trends of soil chemical properties in *C*. *dactylon* and *H*. *altissima* were consistent. Likewise, *T*. *distichum* and *S*. *matsudana* were consistent.

### 3.2. The Soil Bacterial Community Characteristic in Different Periods

**Figure 5:**
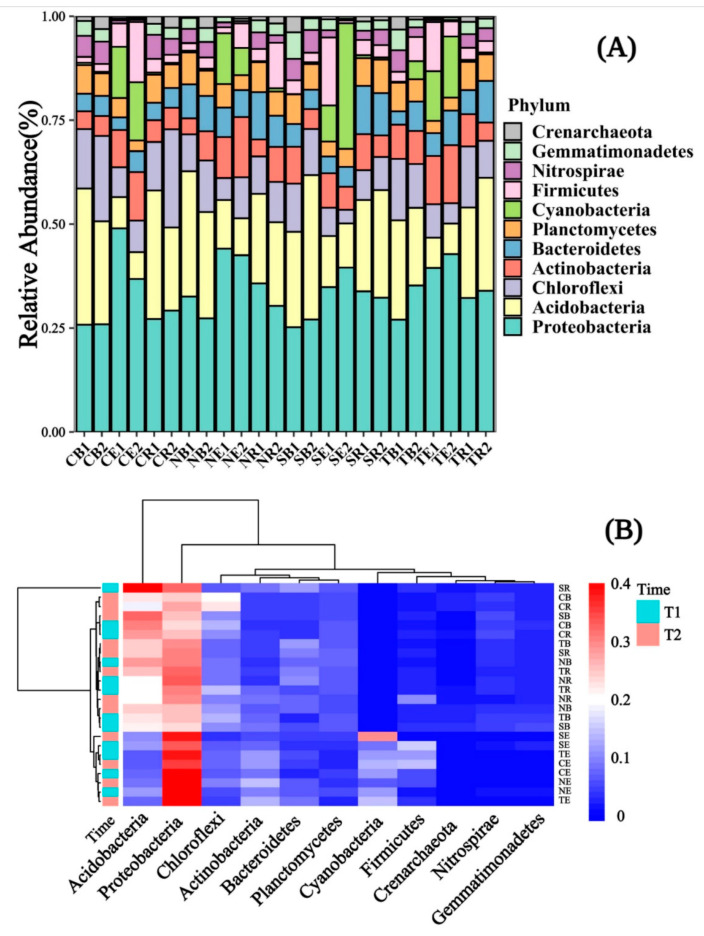
The relative abundance of soil microbial communities was shown with a bar chart and a clustering diagram responding in different emergence phases (T1 for early phase and T2 for late phase) within the riparian zones of the Three Gorges Dam Reservoir, China. Note: Abbreviations in the figures define as T1 (1), T2 (2) for (**A**) only, bulk soil (**B**), rhizosphere soil (R), endophyte (E), *Cynodon dactylon* (C), *Hemarthria altissima* (N), *Salix matsudana* (S), *Taxodium distichum* (T).

The dominant bacteria in the rhizosphere and bulk soil of the selected plants were *Proteobacteria*, *Acidobacteria*, *Actinobacteria*, and *Chloroflexi* (Figure 5A). Still, the dominant bacteria in the endophyte were *Proteobacteria*, *Acidobacteria*, *Actinobacteria*, and *Cyanobacteria*. Moreover, the abundance of *Proteobacteria*, *Actinobacteria*, and *Cyanobacteria* in endophytes was much higher than in the rhizosphere and bulk soils. Conversely, the abundance of *Acidobacteria* was depleted in the endophytes. The relative abundance of *Acidobacteria* and *Chloroflexi* decreased from bulk soil to rhizosphere soil to endophytes, but the relative abundance of *Proteobacteria* and *Actinobacteria* decreased from the endophytes to the rhizosphere to bulk soil. Other bacteria, such as *Bacteroidetes* and *Planctomycetes*, and *Nitrospirae*, in abundance, have little difference across bulk, rhizosphere soil, and endophytes. For the dominant bacteria enriched in endophytes, the *Cyanobacteria* abundance of SE was much higher than that of other selected plants. For the dominant bacteria enriched in the rhizosphere and bulk soil, the *Acidobacteria* abundance of SR was also higher than that of other plants (Figure 5B).

**Figure 6:**
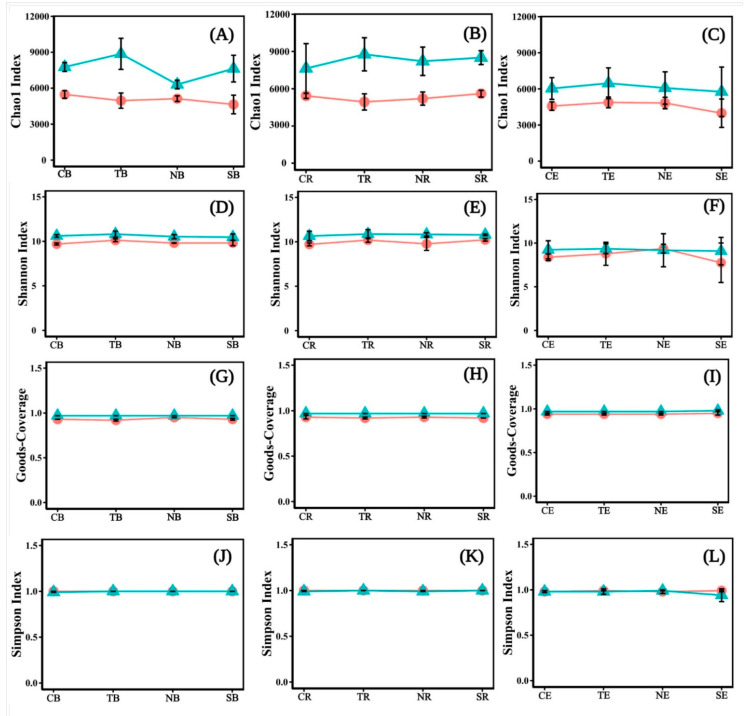
The diversity and richness of bacterial communities were shown with line charts for different emergence phases (T1 for early phase and T2 for late phase) within the riparian zones of the Three Gorges Dam Reservoir, China. The values of bacterial communities were shown by Chao1 Index (**A**–**C**), Shannon Index (**D**–**F**), Goods_coverage (**G**–**I**), and Simpson Index (**J**–**L**). Note: Abbreviations in the figure define as bulk soil (B), rhizosphere soil (R), endophyte (E), *Cynodon dactylon* (C), *Hemarthria altissima* (N), *Salix matsudana* (S), *Taxodium distichum* (T), the early emergence phases (the red line in the figures), the late emergence phases (the green line in the figures).

The Chao1 Index, Shannon Index, Simpson Index, and goods_coverage were used for quantifying bacterial diversity and richness. The Chao1 Index (Figure 6A–C) and Shannon Index (Figure 6D–F) of *T*. *distichum* were higher than those of other plants. The differences between the Chao1 index and goods_coverage (Figure 6G–I) of herbaceous plants in different periods were smaller than those of woody plants, and their values in T2 were relatively higher than in T1. The Shannon index in T2 was higher than in T1, except for NE (Figure 6F). The Simpson Index (Figure 6J,K) was similar to different periods, except that of SE in T1 was higher than in T2 (Figure 6L). The Chao1 index and Shannon index fluctuated more than the Simpson and goods_coverage indices in the same periods. On the whole, we found that the Chao1 Index, Shannon Index, and goods_coverage of the bacteria community in the late emergence phase were higher than in the early emergence phase (Figure 6).

**Figure 7:**
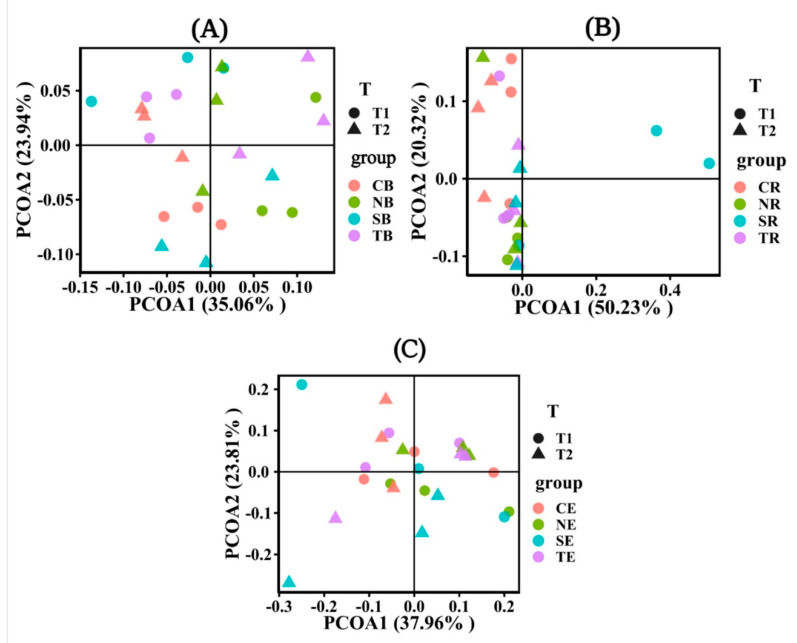
Principal coordinates analysis (PCoA) showed the abundance and diversity of bacterial microbial communities under different emergence phases (T1 for early phase and T2 for late phase) within the riparian zones of the Three Gorges Dam Reservoir, China. The factor loading of PCoA axes 1 and 2 explained 59%, 70.55%, and 61.77% of the total variation for bulk soil in (**A**), rhizosphere soil in (**B**), and endophyte in (**C**). Note: Abbreviations in the figure define as bulk soil (B), rhizosphere soil (R), endophyte (E), *Cynodon dactylon* (C), *Hemarthria altissima* (N), *Salix matsudana* (S), *Taxodium distichum* (T).

In the different emergence periods, the soil bacterial community characteristics for the four plant species differed significantly in bulk soil (Figure 7A). On the contrary, the soil bacterial community characteristics had no significant difference in endophyte and rhizosphere soil in different periods (Figure 7B,C). However, in the rhizosphere (Figure 7B), the soil bacterial community characteristics of *S*. *matsudana* were significantly different from those of other plant soils in T1. Similarly, in the endophyte (Figure 7C), only the bacterial community characteristics of *S*. *matsudana* were significantly different from those of other plants in T2. On the whole, there was no significant difference in the soil bacterial community characteristics among other plants in rhizosphere soil and endophytes (Figure 7B,C). 

### 3.3. Relationship between Soil Microbial Community and Soil Property in Different Periods

**Figure 8:**
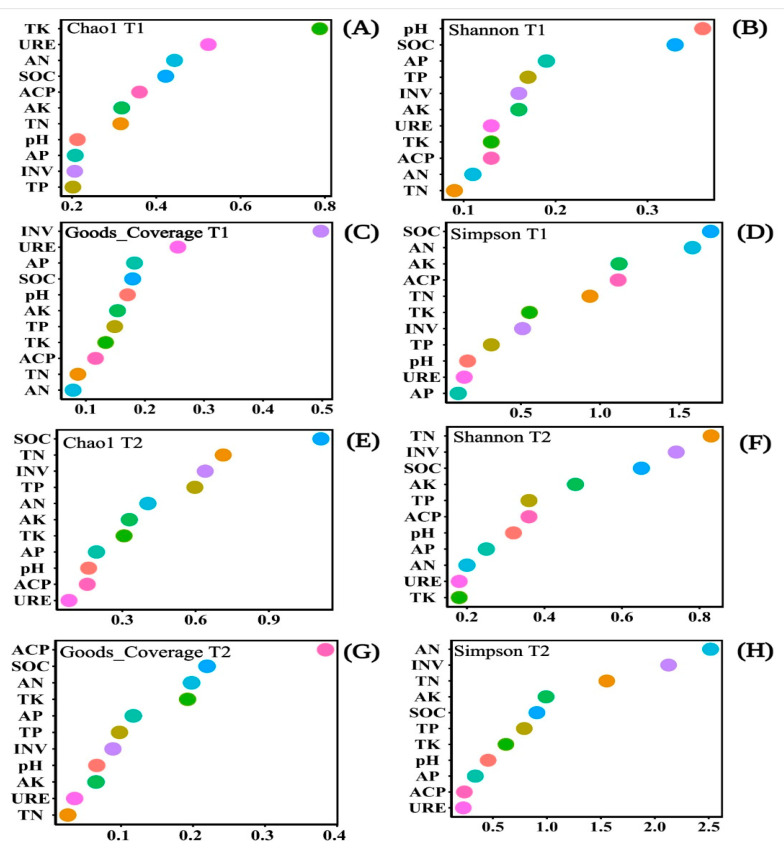
Scattered plots of Random Forest Modeling for the soil chemical properties and soil enzyme activities impacted on the soil bacterial communities shown by the Chao1 index (**A**,**E**), Shannon index (**B**,**F**), Goods_coverage (**C**,**G**), and Simpson index (**D**,**H**) under different emergence phases (T1 for early phase and T2 for late phase) within the riparian zones of the Three Gorges Dam Reservoir, China. The variables were ordered from top-to-bottom, as the most to the least important. Note: Abbreviations in the figure define as pH, available potassium (AK), total potassium (TK), alkali hydrolyzed nitrogen (AN), available phosphorus (AP), soil organic content (SOC), total nitrogen (TN), total phosphorus (TP), acid phosphatase (ACP), invertase (INV), and urease (URE).

The Chao1 index, goods_coverage, Simpson index, and the Shannon index could evaluate the richness and diversity of soil bacterial community characteristics. Soil properties could significantly influence the diversity, evenness, and richness of the bacterial community. The SOC influence on the Chao1 index and goods_coverage index was ranked fourth in T1 (Figure 8A,C). The SOC influence on the Shannon index in T1 and the goods_coverage index in T2 ranked second (Figure 8B,G). The SOC influence on the Simpson index in T1 and the Chao1 index in T2 ranked first (Figure 8D,E). The SOC influence on the Simpson index ranked fifth in T2 (Figure 8H), and the influence of the Shannon Index in T2 ranked third (Figure 8F). The other properties that influence the Chao1 index, goods_coverage, the Shannon index, and the Simpson index, were all unstable. However, the influence of SOC on soil bacterial community characteristics was always at the forefront. Therefore, SOC had an important impact on bacterial community diversity and richness (Figure 8). 

**Figure 9:**
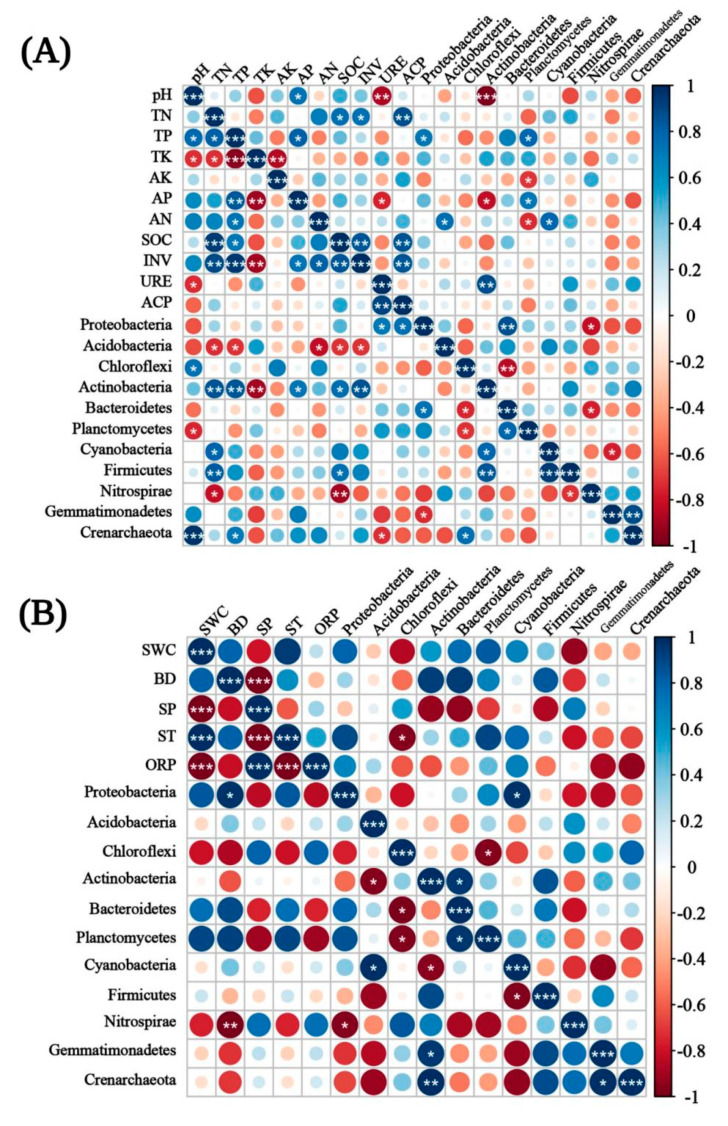
The relationships between soil properties, soil enzyme activities, and soil bacterial communities under different emergence phases (T1 for early phase and T2 for late phase) within the Three Gorges Dam Reservoir, China. The Pearson correlation heat maps show the relationship strengths among soil chemical properties, soil enzyme activities, and bacterial community abundance in (**A**), and among soil physical properties, soil enzyme activities, and bacterial community abundance in (**B**). Circle size and color shade represent the correlation analysis value magnitude and direction. Blue and red represent positive and negative correlations. (The bottom left corner of the figure is T2, and the top right corner is T1). Significant at *p* < 0.001 (***) or *p* < 0.01 (**) or *p* < 0.05 (*).

**Figure 10:**
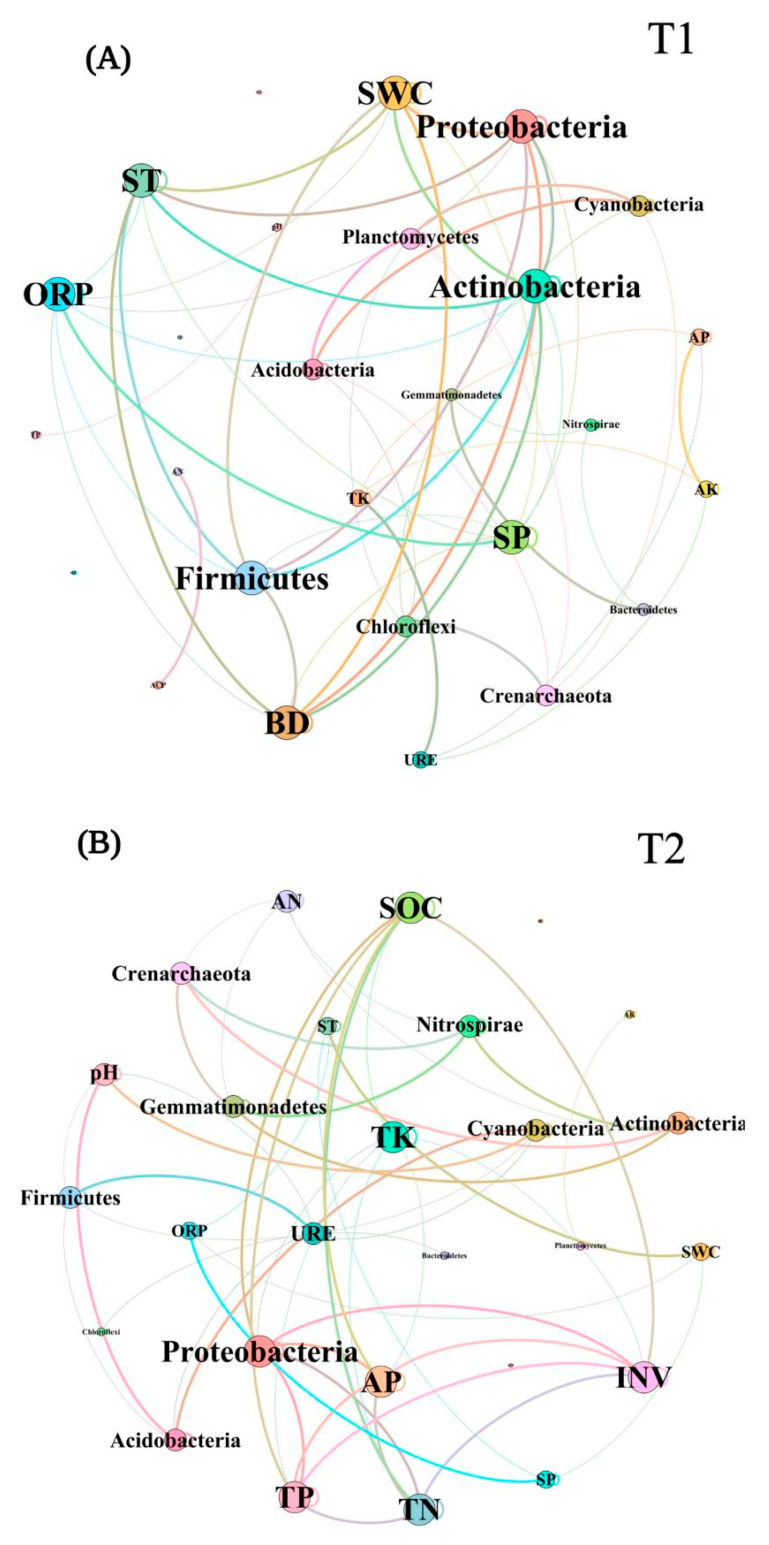
The network topology diagrams showed the weighted degree of the soil properties, soil enzyme activities, and soil bacterial communities in T1 (**A**) and T2 (**B**). The more connections a node has, the higher the weight of the node.

**Figure 11:**
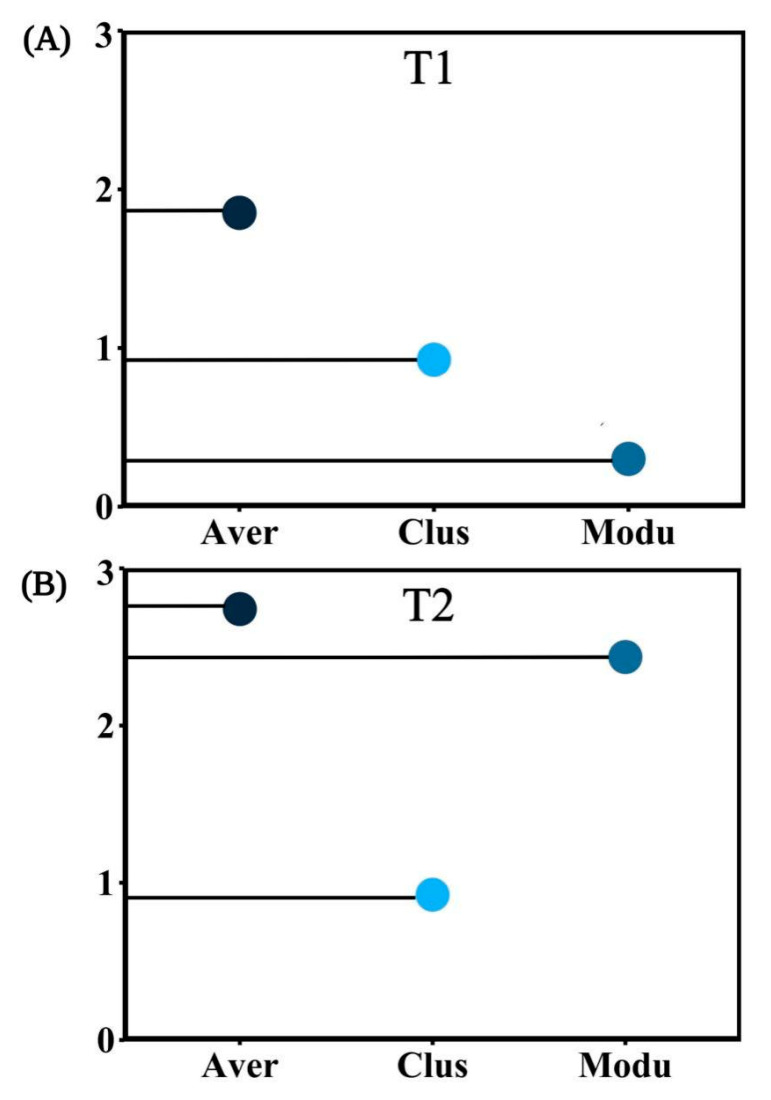
The complexity of T1 (**A**) and T2 (**B**) network topology diagrams. The abbreviations in the figure are defined as Average weighted degree (Aver), Clust Coeff (Clus), Modularity (Modu).

In T1, ACP, AP, and SP were more correlated with the bacterial community, while *Actinobacteria*, *Planctomycetes*, and *Proteobacteria* were significantly correlated with soil enzyme activity and chemical properties. In T2, the bacterial community abundance was significantly correlated with pH, TN, TP, SOC, INV, and BD, while *Actinobacteria*, *Nitrospirae*, *Acidobacteria*, and *Proteobacteria* were significantly correlated with soil enzyme activity and chemical properties (Figure 9A,B). To sum up, *Actinobacteria* and *Proteobacteria* showed a significant correlation with soil properties in different periods. In the co-occurrence networks of soil properties and bacterial communities measured in T1, the weights of SWC, ST, BD, *Proteobacteria*, *Firmicutes*, and *Actinobacteria* were higher than the others (Figure 10A). In T2, however, the weights of SOC, TK, INV, and *Proteobacteria* were higher (Figure 10B). The weight of physical properties was higher in T1, but the weight of chemical properties was higher in T2. Moreover, in T2, the average weighted degree and modularity of co-occurrence networks were higher than in T1, indicating a higher complexity and stability of the soil system (Figure 11A,B).

## 4. Discussion

The present study investigated the plant-soil-microbial interactions at different emergence periods inside the riparian zones of the Three Gorges Dam Reservoir. Overall, our study found that the variation trend of soil properties in *C*. *dactylon* and *H*. *altissima* was consistent in different periods; likewise, *T*. *distichum* and *S*. *matsudana* were consistent (Figure 2, Table 1). The indices indicating the soil properties of *C*. *dactylon* and *H*. *altissima* had relatively high values among the selected plants (Figure 2). Plants with fibrous roots were more effective than those with taproots in reducing soil detachment [43]. In the riparian zones of the Three Gorges Dam Reservoir, high water tables have an impact on the abundance of woody plants rather than herbaceous plants [44,45]. Therefore, herbaceous plants have easier access to soil nutrients in the riparian zone than woody plants, thus benefiting their survival and development [46]. Other studies have shown that the soil nutrient content and tolerance of herbaceous plants were higher than those of woody plants under the stress of flooding or increased rainfall [47,48,49,50]. Yet, the three enzyme activities were highest in T2 (Figure 3), which might be attributed to the temperature that affected soil enzyme activities [51], because the soil temperature was significantly different from T1 and T2 (Table 1). The high temperature in the reservoir area was measured at T1, and water evaporated more extensively at that time, resulting in soil moisture content being one factor for the change of soil enzyme activities. Similarly, higher soil temperatures that inhibit microorganism growth (Figure 6) might also affect soil enzyme release.

Our study also found that only a few bacterial taxa, such as the dominant bacterial-*Proteobacteria*, *Acidobacteria*, *Actinobacteria*, and *Chloroflexi* (Figure 5A), were in the plant rhizosphere and bulk soil. In other reports, these abundant bacterial taxa in the bulk and rhizosphere soil were dominant as well [52,53]. Furthermore, the taxa of *Cyanobacteria* in endophytes were much higher (Figure 5B). However, *Acidobacteria* depleted in the endophyte may be put down to many metabolic pathways related to carbon fixation, aromatic compounds, and amino acid synthesis [54,55,56]. It is also possible that *Acidobacteria* predominates in the leaching environment and are related to the soil pH. When the riparian zone faces a flooded environment, its soil pH deviates from neutral, and the *Acidobacteria* community has more robust systematic clustering [57]. The pH, dissolved nitrogen, and orthophosphate concentrations in roots were higher than in bulk soil [58], promoting *Cyanobacteria* development and growth [59]. Moreover, under flooding stress, *Cyanobacteria* can produce some resistance and help the normal growth and development of plants [60]. Our study also found that bacterial community diversity and evenness were lower in T1 than in T2 (Figure 6). The reason was due to the relatively higher soil temperature in T1, as the soil temperature was significantly correlated with the bacterial community abundance (Figure 9B). High temperatures reduced enzyme activity and were not conducive to microbial growth (Figure 3). This phenomenon happened due to reduced rainfall and water ebb periods in the reservoir area, resulting in the change of soil properties [61]. These were not conducive to the growth and development of microorganisms [62,63]. Additionally, the soil bacterial community abundance of four plants was significantly different from the soil bulk in the different periods (Figure 7A). The endophyte and rhizosphere soil, on the other hand, demonstrated no significant change (Figure 7B,C). Soil nutrients and interactions between endophytic bacteria and cultivars cause histochemical composition differences which result in flora change [64]. Differences in the rhizosphere, inner root layer, and soil microbial community in different pH ranges affected the microbiome composition. The cation exchange capacity and endophytic fungi also dramatically influence soil microbial community formation [65,66]. 

The present study also found that *Actinobacteria* and soil properties showed the strongest correlation (Figure 9A and Figure 10A). *Actinobacteria* are abundant in terrestrial and aquatic ecosystems, especially in soils with highly influenced physiological and metabolic properties. They can decompose humus and play an important role in biogeochemical cycles [67,68], such as the increasing function of *Actinobacteria* as the soils become warmer and drier throughout the world [69]. *Actinobacteria* can also be successfully applied to bioremediation of metal-contaminated soil and water remediation. Its reproduction mode was closely related to chemical and environmental characteristics [70]. We also found that SOC was the main index affecting bacterial community richness and diversity (Figure 8). The SOC mainly comes from vegetation litter, soil microorganisms, soil fauna, and plant root residues [71]. However, all microbial residues contribute to SOC, and bacterial residual carbon contributes more to organic matter than fungus [72]. The addition of organic matter can significantly change the soil microbial community structure and carbon-degrading enzymes, depending on soil improvement and depth. However, the SOC is not affected by the season [73,74]. Moreover, the SOC in the soil can be preserved for a long time, affecting the microbial community by impacting the physical and chemical properties of the soil [75]. In T1, the weight of soil physical properties in the soil co-occurrence networks was higher than that of chemical properties (Figure 10A). In T2, the weight of chemical properties was higher, which led to the soil co-occurrence networks having a higher complexity (Figure 10B). Such phenomena may be because of the change in riparian water level that was reduced slowly in T1. Soils that experience water stress convert NO_3_^−^ to NO_2_^−^, Fe^3+^ to Fe^2+^; the soil pH fails to return to neutral [76,77,78]. The soil chemistry properties are still unstable. On the contrary, after flooding, physical properties such as soil water content, bulk density, and others change [79,80], and high temperatures in T1 control the plant-soil-microbial system. 

While this study provided these plants’ comprehensive bacterial community characteristics and soil property analysis, such research is still in its early stages [81,82,83]. Many studies have shed light on aspects of the plant rhizosphere and bulk soil microbial communities [84,85,86]. However, only by better understanding the soil microbial communities of roots, bulk, and rhizosphere in different artificial vegetation types can we thoroughly investigate a microbiome assembly mechanism for the sustainable management of massive dams and reservoir riparian zones [87,88,89]. In the long term, a better understanding of the relationship between plant microbiota characteristics and soil properties will contribute to microbial strains or consortia and fertilizer to improve the vegetation and soil health and productivity in the riparian zones.

## 5. Conclusions

Our results demonstrated that variations in soil physical and chemical properties in *C*. *dactylon* and *H*. *altissima* were steady during different periods. The indices indicating soil properties of *C*. *dactylon* and *H*. *altissima* had higher values than those of others. The Chao1 Index and Shannon Index in the soil bacterial communities of *T. distichum* were higher than those of other plants. The richness, evenness, and diversity of the bacterial community were mainly affected by soil organic matter. In the early emergence phase, the weight of soil physical properties in the plant-soil-microbial system was higher than that of chemical properties. In contrast, the importance of chemical properties increased in the late emergence phase, and the composition of soil co-occurrence networks was more complex. In different periods, the weight of dominant soil bacteria, including *Proteobacteria*, *Acidobacteria*, *Actinobacteria*, *Chloroflexi*, and *Cyanobacteria*, was higher than in other species, and their composition was different in the rhizosphere, bulk soil, and endophyte. Overall, our research has increased the knowledge of bacteria in bulk and rhizosphere soils, and within roots in different emergence phases. This study suggests that these herbaceous and woody vegetation are suitable for planting in reservoir areas affected by hydrology and human disturbance in light of soil nutrients and soil microbial communities, respectively. These results will help to utilize key microbiota members and fertilizer to improve vegetation and soil health and productivity in the riparian zones of massive dams and reservoirs.

## Figures and Tables

**Table 1:** The contents of soil physical properties under different emergence phases (T1 for early phase and T2 for late phase) within the Three Gorges Dam Reservoir, China. The values of soil water content (SWC), bulk density (BD), soil temperature (ST), oxidation-reduction potential (ORP), and soil porosity (SP) measure in bulk soil for selected plants.

Plant/*p*-Value	SWC/%	BD/g cm^−3^	SP/%	ST/°C	ORP/mv
C/T1	12.29 ± 2.71	1.76 ± 0.06	33.76 ± 2.19	26.87 ± 0.25	326.33 ± 4.51
C/T2	8.01 ± 0.83	1.54 ± 0.01	41.96 ± 0.51	24.17 ± 0.12	375.00 ± 4.58
*p*	ns	*	*	**	**
N/T1	14.5 ± 2.93	1.89 ± 0.04	28.63 ± 1.38	29.47 ± 0.76	296.00 ± 10.58
N/T2	10.00 ± 0.61	1.55 ± 0.16	41.48 ± 5.99	24.37 ± 0.23	307.67 ± 13.65
*p*	*	ns	ns	**	ns
T/T1	13.25 ± 1.12	1.8 ± 0.10	32.03 ± 3.94	29.03 ± 0.85	380.00 ± 14.53
T/T2	7.21 ± 1.19	1.81 ± 0.15	31.7 ± 5.74	22.70 ± 0.26	369.00 ± 21.52
*p*	*	ns	ns	**	ns
S/T1	14.99 ± 0.60	1.83 ± 0.08	31.07 ± 2.99	31.23 ± 0.49	399.67 ± 13.32
S/T2	9.08 ± 2.27	1.77 ± 0.05	33.19 ± 2.05	23.40 ± 0.10	378.33 ± 19.66
*p*	ns	ns	ns	**	ns

**Note:** The significant differences at *p* <  0.01 (**) or *p* <  0.05 (*) according to the paired *t*-tests. Abbreviations in the table define as *Cynodon dactylon* (C), *Hemarthria altissima* (N), *Salix matsudana* (S), *Taxodium distichum* (T).

## Data Availability

The data presented in this study are available in the figure and table.
